# Supplementary material for: Human CD26high T cells elicit tumor immunity against multiple malignancies via enhanced migration and persistence
Source: Nat Commun. 2017 Dec 6;8:1961. doi: 10.1038/s41467-017-01867-9 (PMC5719008; doi:10.1038/s41467-017-01867-9)
Supplement: Supplementary file 1 — Supplementary Information [file 41467_2017_1867_MOESM1_ESM.pdf]

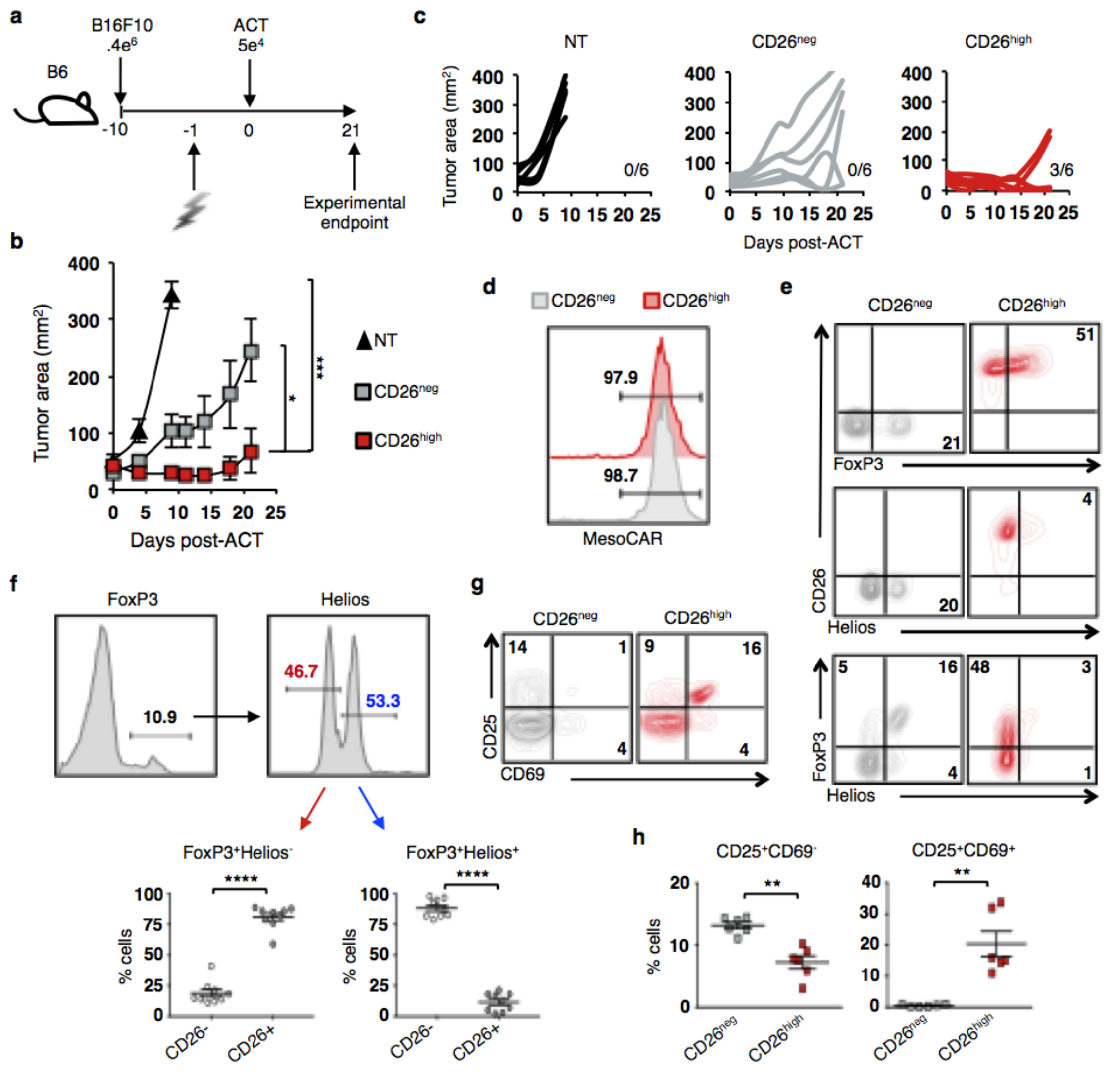

Supplementary Figure 1. CD26^{high} T cells are activated and regress malignancy to a greater extent than CD26^{neg} T cells. Splenocytes from transgenic TRP-1 mice were isolated and cultured with 1µl/ml TRP-1 peptide and feeder cells at a ratio of 1 feeder:5 TRP-1 cells. TRP-1 CD4^{+} T cells were programmed to a Th17 phenotype using polarizing cytokines (10ng/ml hIL-1β, 100ng/ml hIL-21, 100ng/ml hIL-6, 30ng/ml hTGFβ, 10µg/ml αm-IFNγ, 10µg/ml αm-IL-4) and expanded with 100IU/ml IL-2. After six days expansion, Vβ14^{+}CD4^{+} Th17 cells were sorted by CD26 into CD26^{neg} (bottom ~10%) and CD26^{high} (top ~5%). **a-c**, B6 mice bearing B16F10 melanoma established for 10 days were left untreated (NT; No Treatment) or infused with 5e^{4} CD4^{+}Vβ14^{+} CD26^{neg} or CD26^{high} T cells. All mice were lymphodepleted with 5 Gy total body irradiation one day prior to ACT (N=6 mice/group, representative of two individual experiments). P values for the tumor curve were calculated using One-way ANOVA with a Kruskal-Wallis comparison on the last day that all mice from compared groups were alive (NT vs. CD26^{high}=day 9, CD26^{neg} vs. CD26^{high}=day 21). **d**, Representative histograms of human CD26^{neg} and CD26^{high} T cells after being sorted for MesoCAR expression, yielding ~98% pure population **e**, Representative FACS plots of transcription factors from sorted human CD4^{+} T cells prior to bead stimulation. **f** Summary graphs of CD26 expression on CD4^{+}FoxP3^{+}Helios^{-} and CD4^{+}FoxP3^{+}Helios^{+} T cells using back gating on FlowJo

software from the bulk $CD4^+$ T cell subset. **g** and **h**, Representative FACS plots (**g**) and summary data (**h**) of activation markers from six individual donors. One-way ANOVA with Kruskal-Wallis comparison was performed on **f** and **h**. Data with error bars represent mean ± SEM. *, P < 0.05; **, P < 0.01.; ***, P < 0.001; ****, P < 0.0001.

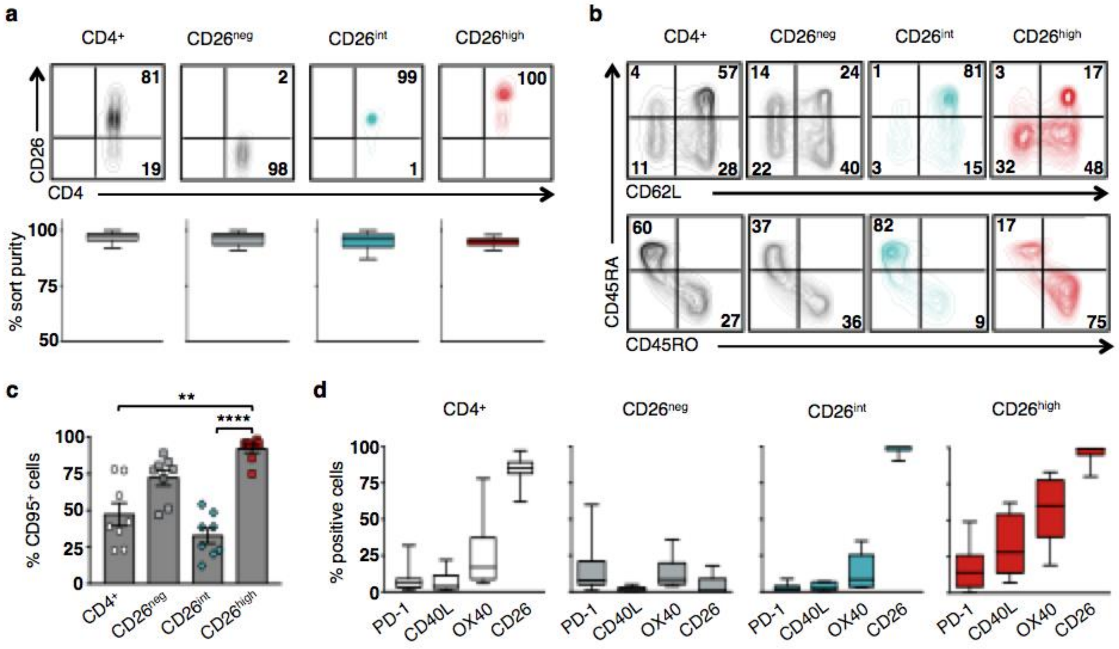

Supplementary Figure 2. CD26-sorted subsets are pure and have unique profiles. Human CD4$^+$ T cells from healthy donors were sorted by CD26 expression. **a**, Post-sort phenotype (top, representative of 26 donors) and purity (bottom, N=26) of all sorted subsets was determined using flow cytometry. **b-d**, Representative FACS plots of memory (**b**; N=26) and graphical representation of CD95 expression (**c**; N=8) or co-costimulatory/co-inhibitory markers (**d**; N=8-12) by flow cytometry prior to cell activation. One-way ANOVA with Kruskal-Wallis comparison was performed on **c**. Data represented as mean ± SEM. **, P < 0.01; ****, P < 0.0001.

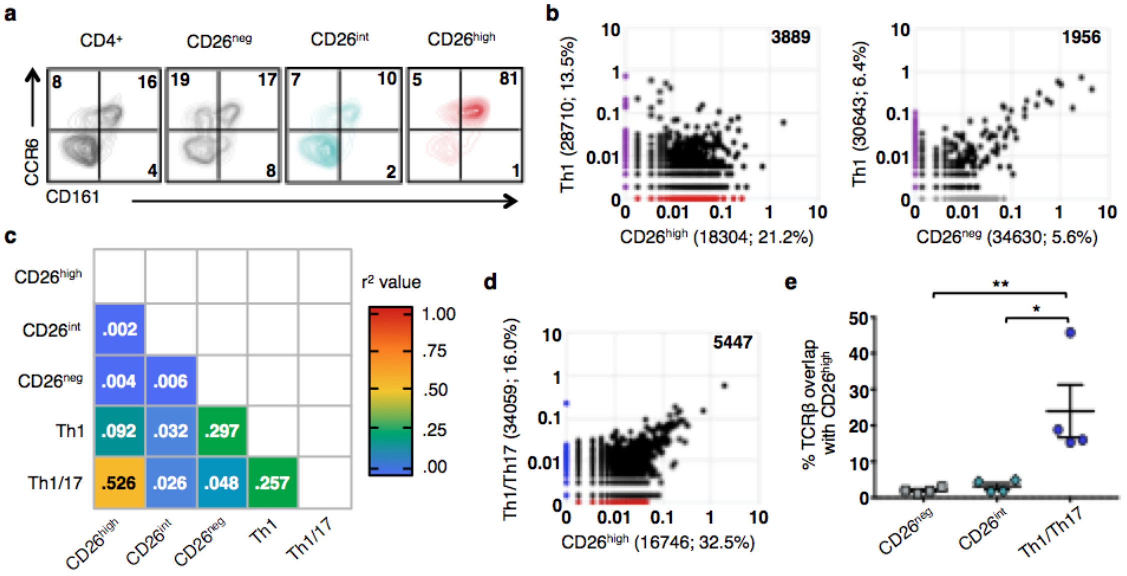

Supplementary Figure 3. TCRβ sequences of CD26^{high} T cells overlap with Th1/Th17 cells, but not Th2. a, Human CD4^{+} T cells were sorted by CD26 and analyzed for CCR6 and CD161 expression by flow cytometry (representative of 26 donors). Human CD4^{+} T cells were sorted into bulk CD4^{+}, CD26^{neg}, CD26^{int}, CD26^{high}, Th1 (CXCR3^{+}CCR6^{-}), Th2 (CCR4^{+}CCR6^{-}), Th17 (CCR6^{+}CCR4^{+}) and Th1/Th17 (CXCR3^{+}CCR6^{+}). DNA was isolated from sorted subsets and TCRβ sequencing was performed using an immunoSEQ kit and subsequent analysis. **b**, Graphical representations of TCR overlap between Th1 cells and CD26^{high} or CD26^{neg} (N=4). **c**, Correlation of TCR expression (r^2) determined and graphed using a heatmap diagram (N=4). **d** and **e**, TCR overlap between Th1/Th17 and CD26^{high} (**d**) and CD26^{high} compared to CD26^{neg}, CD26^{int} and Th1/Th17 cells (**e**) was defined via immunoSEQ software (N=4). One-way ANOVA with Kruskal-Wallis analysis was performed on **e**. Data represented as mean ± SEM. *, P < 0.05, **, P < 0.01.

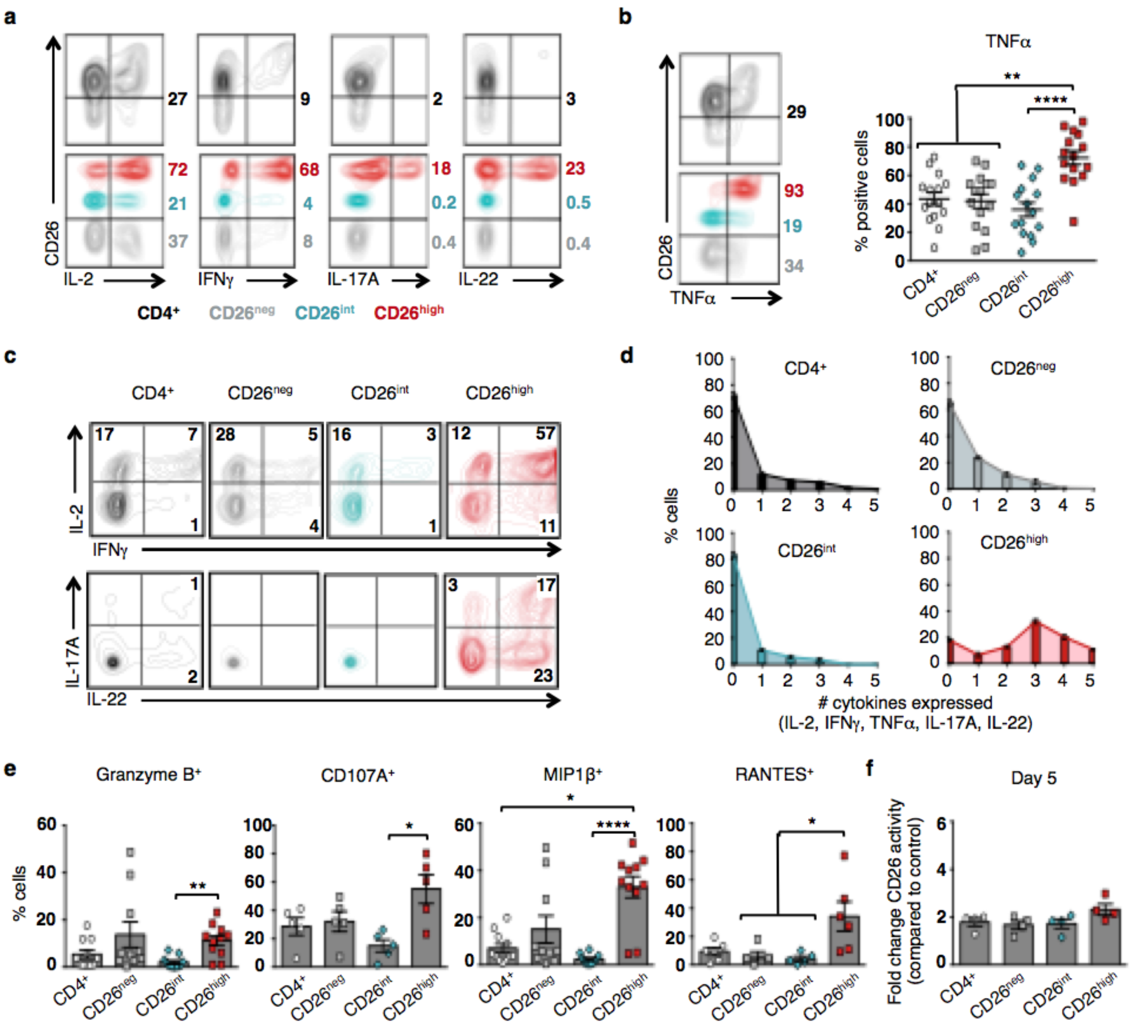

Supplementary Figure 4. CD26^{high} T cells are highly functional and cytotoxic. CD4^{+} T cells from the blood of healthy individuals were sorted by CD26 expression (**Fig. 2A**). **a-e**, Sorted cells were activated with PMA/Ionomycin and Monensin for 4 hours prior to intracellular staining (N=5-26). In **d**, three independent donors were analyzed by FlowJo software and graphed to display the percentage of cells simultaneously secreting 0-5 cytokines (IL-2, IFNγ, TNFα, IL-17A, IL-22). **f**, 1e^{5} sorted cells were incubated with the CD26 ligand gly-pro-P-nitroanalide at 37°C for 2 hours and enzyme activity was determined using a colorimetric assay (N=5). One-way ANOVA with Kruskal-Wallis comparison was performed on **b**, **e** and **f**. Data with error bars represent mean ± SEM. *, $P < 0.05$; **, $P < 0.01$; ****, $P < 0.0001$.

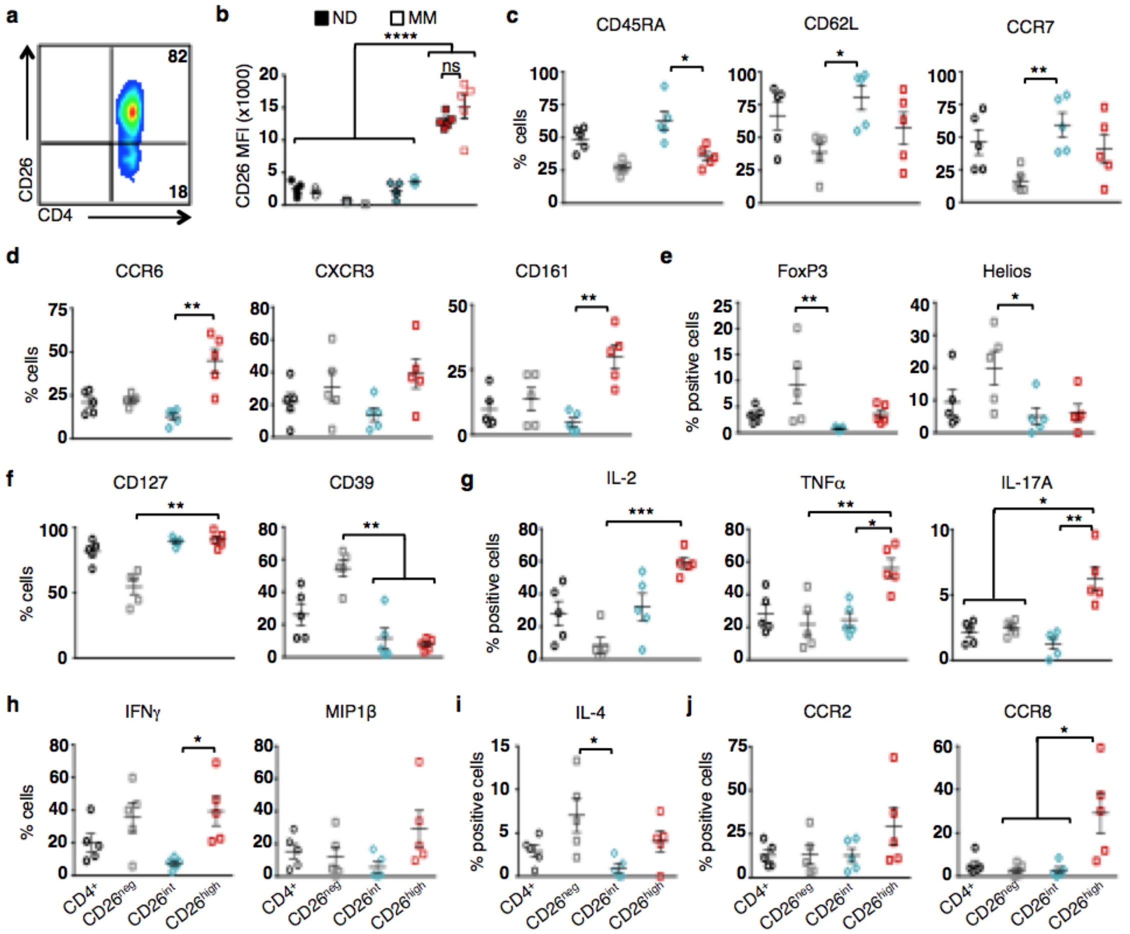

Supplementary Figure 5. CD26-sorted subsets in patients with metastatic melanoma have a similar functional and phenotypic profile as those enriched from healthy donors. The biologic properties of CD26^{neg}, CD26^{int} and CD26^{neg} T cells from the blood of patients with metastatic melanoma were analyzed via flow cytometry (N=5). **a**, Representative FACS plot of CD26 expression on CD4^{+} T cells. **b**, Graphical representation of CD26 MFI on sorted T cells from cancer patients (N=5) and healthy individuals (N=5). **c-j**, Scatter plots of sorted T cells from melanoma patients analyzed by flow cytometry. Intracellular stains were performed following activation with PMA/Ionomycin and Monensin for four hours (N=5). One-way ANOVA with Kruskal-Wallis comparison was performed for **b-j**. Data represents mean ± SEM. *, $P < 0.05$; **, $P < 0.01$; ***, $P < 0.001$; ****, $P < 0.0001$.

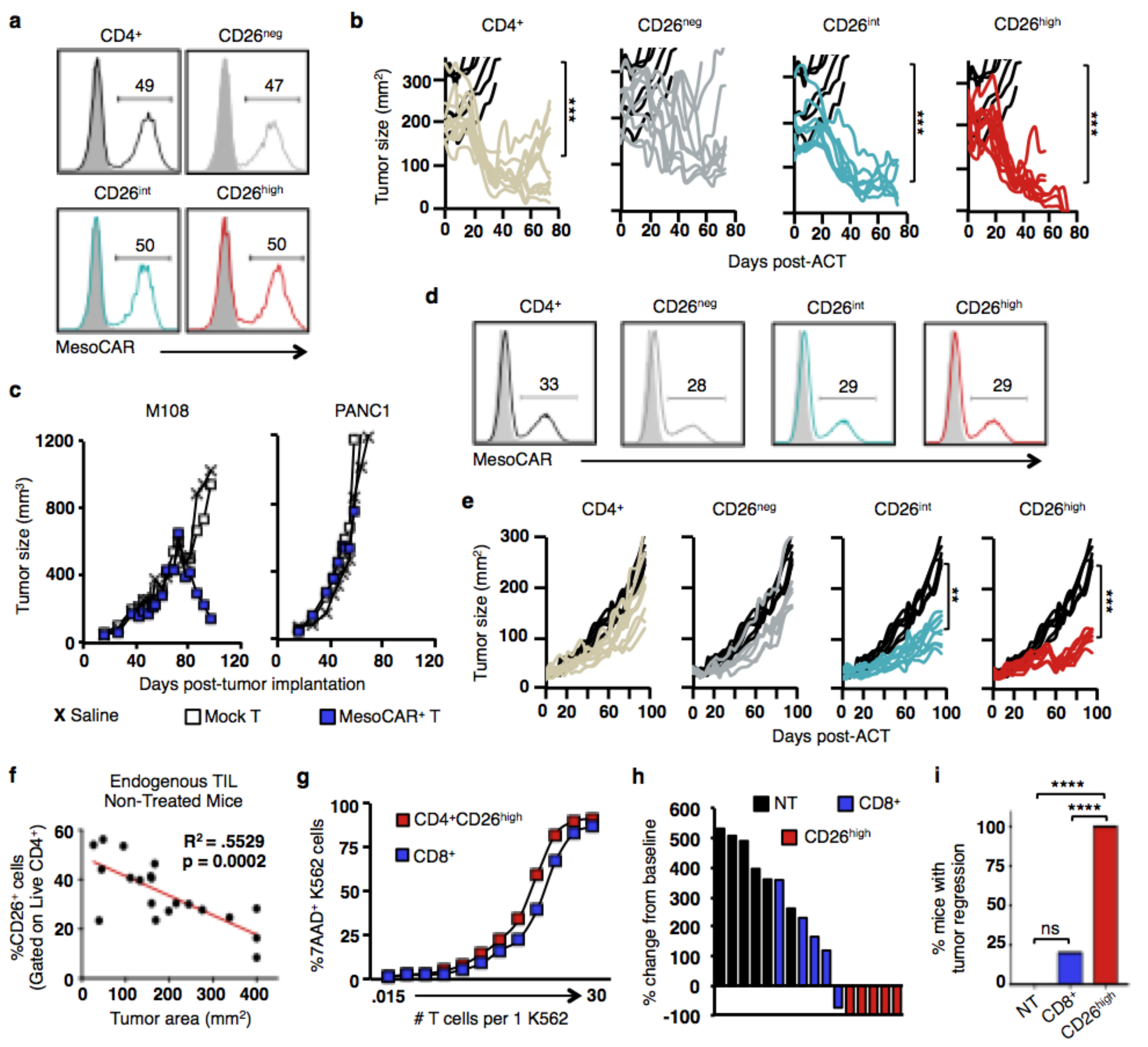

Supplementary Figure 6. CD26^{int} and CD26^{high} T cells regress multiple tumors. CD4^{+} lymphocytes from healthy donors were sorted by CD26 expression and stimulated with CD3/ICOS beads (1 bead : 5 T cells). Cells were transduced at 36 hours post-activation to express a 1st generation mesothelin-specific CD3ζ CAR and expanded in IL-2 (100IU/ml) for 10 days. **a**, MesoCAR transduction efficiency was determined using flow cytometry prior to ACT. **b**, Individual tumor curves for each treatment group compared to no treatment (black lines) for M108-bearing NSG mice discussed in Fig. 5 (N=7-9 mice/group). P values for tumor curves were calculated by Mann-Whitney on the final day when mice from all comparison groups were still alive (NT vs. all groups=day 38). **c**, NSG mice bearing either mesothelioma or pancreatic cancer were infused with 12.5e^{6} non-specific mock T cells or 12.5e^{6} transduced T cells (~40% MesoCAR^{+}) on days 49 (PANC1) or 75 (M108) post-tumor inoculation. **d**, MesoCAR transduction efficiency of sorted T cell subsets were determined by flow cytometry prior to ACT into

PANC1-bearing mice. **e**, Individual tumor curves for each treatment group compared to no treatment (black lines) for NSG mice with pancreatic cancer (6-9 mice/group). P values for tumor curves were calculated by Mann-Whitney on the final day when mice from all comparison groups were still alive (All groups=day 84). **f**, TIL were isolated from un-treated, melanoma-bearing mice and assessed for CD26 expression via flow cytometry (N=20). **g-i**, MesoCAR$^+$ CD4$^+$CD26^{high} and CD8$^+$ T cells were assessed for their ability to eradicate cancer cells *in vitro* (**g**) and *in vivo* (**h-i,** N=6 mice/group). Data in **h** and **i** represent 60 days post-ACT. One-way ANOVA with Kruskal-Wallis component was performed on **i**. Data with error bars represent mean ± SEM. **, $P < 0.01$; ***, $P < 0.001$; ****, $P < 0.0001$.

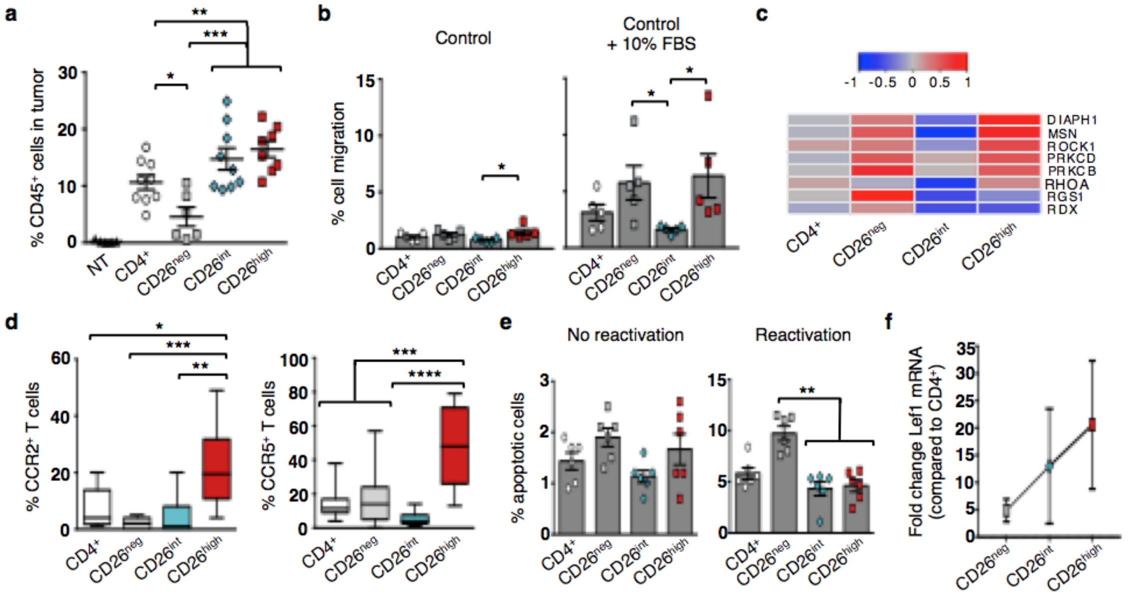

Supplementary Figure 7. Stem memory CD26^{high} T cells persist and migrate to the tumor. a, Tumors from PANC1-bearing mice were harvested and the percentage of CD45$^+$ T cells was determined by flow cytometry (N=7-9). **b**, 7.5e4 bulk CD4$^+$, CD26^{neg}, CD26^{int} and CD26^{high} T cells were assayed for migration towards control media (top) and media supplemented with 10% FBS (bottom) using a transwell assay (N=5). **c**, Expression of migration-related genes in pre-activated, sorted T cells was determined via gene array and graphed as a (+/-) log2-fold change (N=3-5). **d**, Chemokine receptor expression on inactivated, sorted T cells was assessed via flow cytometry (N=15). **e**, Ten-day expanded T cells were analyzed for apoptosis using Annexin V/PI staining in the presence or absence of overnight activation with anti-CD3 antibody (N=6). **f**, Lef1 expression in sorted T cell subsets was determined by qPCR analysis (N=4). One-way ANOVA with Kruskal-Wallis comparison was performed for **a**, **b** and **d-j**. Data represents mean ± SEM. *, $P < 0.05$; **, $P < 0.01$; ***, $P < 0.001$; ****, $P < 0.0001$.

Target	Clone	Fluorophore	Provider
**αm-CD4**	GK1.5	APC Cy7	BD Biosciences
**αm-Vβ14**	14.2	FITC	BD Biosciences
**αm-CD26**	H194-112	PE	BioLegend
**αh-CD26**	C5a5b	PE	
**αh-CD4**	OKT4	APC Cy 7	
**αh-CD25**	BC96	FITC	
**αh-CD45RA**	HI10U	PerCP Cy5.5	
**αh-CD45RO**	UCHL1	APC	
**αh-OX40**	ACT35	PE Cy7	
**αh-CD154/CD40L**	24-31	AF488	
**αh-CCR6**	G034E3	PE Cy7	
**αh-CXCR3**	G025H7	BV421	
**αh-CD127**	A019D5	PE Cy7	
**αh-CD39**	A1	PE Cy7	
**αh-CD45**	HI30	Pacific Blue	
**αh-FoxP3**	150D	AF647	
**αh-Helios**	22F6	Helios	
**αh-IL-22**	516406	AF647	
**αh-TNFα**	MAb11	PE Cy7	
**αh-Granzyme B**	515403	FITC	
**αh-RANTES**	VL1	PerCP Cy5.5	
**αh-CD4**	RPAT4	V500	BD Biosciences
**αh-PD1**	M1H4	FITC	
**αh-CD28**	CD28.2	APC H7	
**αh-CCR7**		PE Cy7	
**αh-CCR2**	48607	AF647	
**αh-CCR5**	2D7	V450	
**αh-IL-2**	MQ1-17H12	PE Cy7	
**αh-IFNγ**	B27	V450	
**αh-IL-17A**	N49-653	PerCP Cy5.5	
**αh-CD107A**	H4A3	APC H7	
**αh-MIP1β**		PerCP Cy5.5	
**αh-IL-4**		FITC	
**Streptavidin**		V500	
**αh-CD69**	FNS0	APC	eBioscience
**αh-ICOS**	ISA-3	Biotin	
**αh-CD161**	HP-3G10	PerCP Cy5.5	
**αh-CCR4**		FITC	R&D Industries
**αh-CCR8**		PerCP Cy5.5	R&D Industries

Supplementary Table 1. Complete list of flow cytometry antibodies.
